# Identifying prognostic indicators for cognitive stimulation therapy for dementia: protocol for a systematic review and individual participant data meta-analysis

**DOI:** 10.1192/bjo.2023.46

**Published:** 2023-04-17

**Authors:** Dominic Crawley, Rob Saunders, Joshua E. J. Buckman, Esther Hui, Richard Walker, Catherine Dotchin, Aimee Spector

**Affiliations:** Institute of Health and Society, Newcastle University, UK; Department of Clinical, Educational and Health Psychology, University College London, UK

**Keywords:** Dementia, psychosocial interventions, statistical methodology, randomised controlled trial, outcome studies

## Abstract

**Background:**

Cognitive stimulation therapy (CST) is the only non-pharmacological, treatment for dementia recommended by the UK National Institute for Health and Care Excellence, following multiple international trials demonstrating beneficial cognitive outcomes in people with mild-to-moderate dementia. However, there is limited understanding of whether treatment prognosis is influenced by sociodemographic and clinical variables (such as dementia subtype and gender), information which could inform clinical decision-making.

**Aim:**

We describe the protocol for a systematic review and individual patient data meta-analysis assessing the prognostic factors related to CST. In publishing this protocol, we hope to increase the transparency of our work, and keep healthcare professionals aware of the latest evidence for effective CST.

**Method:**

A systematic review will be conducted with searches of the bibliographic databases Medline, EMBASE and PsycINFO, from inception to 7 February 2023. Studies will be included if they are clinical trials of CST, use the Alzheimer's Disease Assessment Scale – Cognitive Subscale (gold-standard measure of cognition in dementia in clinical trials) and include participants with mild-to-moderate dementia. Following harmonisation of the data-set, mixed-effect models will be constructed to explore the relationship between the prognostic indicators and change scores post-treatment.

**Conclusions:**

This is the first individual patient data meta-analyses on CST, and has the potential to significantly optimise patient care. Previous analyses suggest people with advanced dementia could benefit more from CST treatment. Given that CST is currently used post-diagnosis in people with mild-to-moderate dementia, the implications of confirming this finding, among identifying other prognostic indicators, are profound.

## Background

Dementia is a clinical syndrome characterised by declining cognition and functional impairment.^1^ Globally, the prevalence of dementia is expected to rapidly increase, and by 2050, there could be 152.8 (95% uncertainty interval 130.8–175.9) million people living with dementia worldwide.^2^ Much of this growth is anticipated to occur in North Africa and the Middle East, where the prevalence of dementia is expected to rise by 367% (95% uncertainty interval 329–403), and eastern Sub-Saharan Africa, where the prevalence is expected to rise by 357% (95% uncertainty interval 323–395). This is likely a result of demographic transition and population growth.^2^ The growing dementia prevalence and the geographic disparity in dementia incidence highlight the necessity for cost-effective, easily delivered interventions that effectively reduce cognitive decline and functional impairment.

Cognitive stimulation therapy (CST) is an evidence-based treatment that has demonstrated beneficial cognitive outcomes (improved cognition and reduced cognitive decline) in persons with mild-to-moderate dementia in multiple international trials.^3,4^ CST follows a format of 14 sessions over 7 weeks, with enrolment immediately post-diagnosis.^3^ Participants are grouped according to baseline cognitive performance (to facilitate maximal engagement with other participants) and partake in games and discussions around a variety of session topics, which have been designed to alleviate symptoms of dementia through stimulation of neuroplasticity.^5,6^ As a non-specialist intervention, any health or social care professional can be trained to deliver CST, and there is good evidence of its cost-effectiveness.^7–9^

## Review rationale

Although many studies have shown positive outcomes following CST, there is limited understanding of whether treatment efficacy is dependent upon individual participant characteristics, including sociodemographic and clinical variables. Prognostic research helps to inform clinical decision-making and optimise patient care.^10^ Previous clinical trials have identified several participant characteristics indicative of CST treatment outcomes; however, to date, most prognostic analysis into CST has been limited by low power.^11–13^ One previous, higher-powered, pre–post analysis identified several characteristics (including female gender and older age) as factors associated with better post-treatment cognitive performance.^14^ Further research, aggregating data across studies to achieve greater power, is required to provide stronger evidence for the potential prognostic factors for CST.

Individual participant data (IPD) meta-analysis is an increasingly used method for aggregating data from multiple sources; instead of extracting summary statistics from relevant publications, it involves sourcing original participant data from suitable studies. This approach is the new gold standard for treatment efficacy research and has recently been used to identify prognostic factors from a range of treatments.^15,16^ IPD meta-analysis achieves greater statistical power than conventional meta-analysis, has the advantage of being able to control for available confounding factors, overcomes a number of sources of bias applicable to meta-analyses of aggregate data and, if a large proportion of eligible studies are able to be included, findings may be more generalisable.^17^ The aims of the study are: (a) to identify all studies exploring the effectiveness of CST through a systematic review; (b) to collect IPD from study authors and create a pooled CST database and (c) to identify prognostic factors associated with outcomes from CST.

## Method

This protocol is being published after search strings have been developed and scoping searches have been performed on a single database (Medline) (to include entries on that database up until 7 February 2023). Formal searches will be undertaken once the protocol has been published, to avoid introducing bias. We originally intended to carry out formal searches toward the end of 2021, but experienced delays after registering the protocol with the International Prospective Register of Systematic Reviews (PROSPERO; registration number CRD42021285440). These are detailed within the discussion. The original protocol included the phrase, ‘ongoing for several months’ to give the reader a sense of the publication timeframe that we have been working toward; however, on review, we appreciate that it was not specific enough to outline the process undertaken. The dates within the revised protocol are now accurate.

### Search strategy

Studies will be identified with the following bibliographic libraries: Medline (1996 to 7 February 2023), EMBASE (1996 to 7 February 2023) and PsycINFO (2002 to 7 February 2023). A final search will be undertaken just before publication of the results, to ensure no studies published more recently have been missed. Keywords related to CST and subject headings related to dementia were combined with the AND and OR Boolean terms, to increase hits. The final search will be limited to full texts in English, published in peer-reviewed journals. A full list of search terms used to identify studies can be found in Supplementary Table 1 available at https://doi.org/10.1192/bjo.2023.46. Two relevant systematic reviews on CST have been identified during scoping searches, and the reference lists of these papers will be manually searched and experts in the field will be consulted about further potentially relevant studies.^18^ Titles and abstracts of potential studies will then be considered against inclusion criteria and read in full by two reviewers (D.C. and E.H.) for potential suitability. A consensus meeting with the entire research team will be carried out to discuss any disagreements between these two appraisers.

### Inclusion criteria

Studies will be included if they: (a) are clinical trials (this includes randomised controlled trials (RCTs), pseudo-RCTs, non-RCTs, and feasibility and pilot trials); (b) used the 14-session CST protocol;^3^ (c) used the Alzheimer's Disease Assessment Scale – Cognitive Subscale (ADAS-Cog) as the cognitive outcome measure; and (d) included participants with mild-to-moderate dementia.

### Data extraction and management

Data will be required and provided through direct contact with the study authors, unless they are held on publicly available repositories. Once studies have been identified for suitability, authors will be contacted using a secure university-registered email address with multifactor authentication. Sets of IPD will be stored on a password-locked computer. Once individual participant data-sets have been pooled together, integrity checks will be carried out to ensure all errors in transcribing data are identified and discussed with chief investigators.^19^ Primary analysis of each publication will be recreated and compared to end-point values from the original publication.

### Risk of bias

Assessing risk of bias of will be handled at the study level. As recommended for systematic reviews of prognostic studies, two researchers (D.C. and E.H.) will independently assess the risk of bias of included studies, using the Quality in Prognosis Studies (QUIPS) appraisal tool.^20^ The QUIPS is used to identify potential bias in six domains: study participation, study attrition, prognostic factor measurement, outcome measurement, study confounding, and statistical analysis and reporting. A three-grade scale (high/moderate/low) is then used to determine the risk of bias in each domain, following discussion between appraisers to reach consensus. Disagreements between appraisers will then be resolved with a consensus meeting with the whole research team. Hayden et al (who developed the QUIPS tool) recommend against aggregating an overall risk-of-bias score, and as such, scores for individual domains will be reported.^20^ Risk of bias will be controlled for by recreating the analysis with each included study removed. Studies found to be at high risk of bias will be removed (individually) from the subgroup analysis. The same will be performed for studies that contribute significantly to heterogeneity.

### Selection of prognostic indicators

We intend to include all prognostic indicators that may influence CST outcomes and that are available in a sufficient number of included studies. Any demographic or clinical variable that can be analysed with a power of 0.8 will be included. Based on initial literature scoping, we anticipate that participant-specific clinical and sociodemographic variables, including age, gender, dementia subtype and initial dementia severity, may be likely factors, but all possible variables will be considered. Dementia severity (measured by baseline cognition) will be of particular interest, given that CST is currently used post-diagnosis for people with mild-to-moderate dementia. Interestingly, some previous analyses have shown greater improvements in ADAS-Cog scores in people with worse cognitive function to begin with; however, this is yet to be reliably replicated.^3^ The present analysis has the potential to completely overhaul current treatment approach and lead to significant optimisation of patient care.

### Outcome measures

The ADAS-Cog has been selected as the cognition outcome measure as it is considered the gold-standard measure of cognition in dementia trials, enabling comparison of dementia interventions.^21^ Developed in the 1980s, the ADAS-Cog comprises three subscales (language, memory and praxis) and has an overall score of 70, with higher scores indicating poorer cognitive performance.^22,23^ The tool also appears to be the most consistently used cognitive outcome measure in trials of CST, and has been translated into several languages and cross-culturally validated in several settings, which should maximise the number of studies eligible for this analysis.^11,24–28^ In trials of multiple psychotherapies, only outcomes from CST will be analysed; moderation against other interventions will not be considered.

### Data analysis

A one-stage, meta-analytic approach will be utilised because we anticipate several of our included studies to have small sample sizes, and the one stage approach is recommended in these situations.^29^ This approach also allows for more flexibility for complex modelling when investigating associations.^17^ After harmonisation, mixed-effect linear regression models will be constructed, with the ADAS-Cog score used as the dependent variable and available prognostic indicators included as independent variables. Study identification will be added as a random effect to account for data clustering. Dummy variables will be created for categorical variables. Multiple imputation using multivariate imputation by chained equations will be used to account for missing data, with multilevel imputation models considered if there is systematic missingness of variables by study. Sensitivity analysis will be performed only on observations with complete data, to assess the impact of the imputation.

The levels and patterns of missing data will be explored and described, and potential missing mechanisms will also be explored, comparing complete cases to those with missing data on any variables used for these analyses. Complete-case analysis will be used if the proportion of missing data are <5% and if the impact of the missing data is negligible. If the impact of missing outcome data appears unlikely to be negligible and there is informative missingness (i.e. data are not missing at random and therefore dependent on the sociodemographic and clinical variables), then we will compute best–worst and worst–best case scenarios to handle missingness in sensitivity analyses. A best–worst case scenario data-set would be generated with the assumption that all participants lost to follow-up in one group would have reported better cognition at the primary study end-point, and all those with missing outcomes in the other group would have reported a decline in cognition. The converse would be used to simulate a worst–best case scenario for sensitivity analyses. If data are missing at random and there is >5% missingness, then missing data will be imputed using multiple imputation with chained equations with ‘MI Impute’ in Stata version 16 for Windows.^31^ Imputation models will be run individually in each study including all variables with <50% missingness, and will be run to give 50 imputed data-sets before appending the imputed study data-sets together for the analyses. The effect of the imputation will be checked in sensitivity analyses run with cases with complete data only.

Heterogeneity between randomised and non-randomised trials will be explored and controlled for. We will also consider whether included studies had similar cohorts of participants, and whether different studies excluded participants with certain sociodemographic and clinical variables. The degree of heterogeneity will be assessed using prediction intervals and its impact assessed with the *I*^2^ statistic.^30^ Stata version 16 will be used for analysis.

### Ethics and dissemination

Once data analysis is complete, findings will be written up for peer-review publication and disseminated accordingly.

To ensure this analysis meets appropriate ethical standards, we will only use data from studies granted ethical approval from an appropriate ethics committee. As this analysis will use data collected from human participants, the research team will enquire with data custodians from eligible studies to ensure that all participants whom we collect data for gave their consent for enrolment. Ethics approval was not required as this meta-analysis did not involve primary data collection. To ensure anonymity, all authors will be asked to remove any identifiable participant information before transfer. A secure, university-registered email address with multifactor authentication will be used to contact authors and handle participant data.

### Patient and public involvement

Neither patients nor the public are involved in the design, conduct, reporting or dissemination of plans for our research. Patients or the public (a person with dementia or a carer) will be invited to the team when interpreting the results, specifically considering implications for clinical practice.

## Discussion

### Study status

At the time of writing, scoping searches have been performed to identify key ‘marker’ studies that very clearly meet all inclusion criteria and that we know must be found in the final searches for those searches to be valid. As such, some studies in the scoping search were read in full. A final search will be carried out just before the publication of the results. Since this review was first registered on PROSPERO in Autumn 2021, there have been some minor amendments to the protocol. First, there has been a change in authorship. Esther Hui has replaced Sarah Hoare, who was unable to continue with the review. Second, the anticipated completion date has been extended to allow sufficient time for the protocol to undergo peer review. In addition, our last search is planned for 2023 rather than 5 December 2021. Finally, when the protocol was registered on PROSPERO, a mistake was made in the type of study to be included. ‘Pre-/post-analysis' was amended to ‘non-randomised controlled trials’ to better describe the type of study to be included, in terms more familiar to readers. Once all eligible studies have been identified, contacting principal authors, sourcing IPD and data analysis will then follow. We used the Preferred Reporting Items for Systematic Reviews and Meta-Analyses Protocols (PRISMA-P) checklist to write this protocol (Supplementary Table 2), and we will conform to the PRISMA guidelines for the development of the systematic review.^32^

### Clinical implications of research

CST is currently offered as a post-diagnostic treatment for people with early-stage dementia.^33^ In the original CST trial, however, most participants had moderate-stage dementia (ADAS-Cog mean score of 27.4, s.d. 2.7), and treatment response in this cohort was considerable (mean change difference between treatment group and control, 2.37; s.d. 0.87; *P* = 0.014).^3^ Baseline cognitive performance has varied between different CST cohorts, as has response to treatment, suggesting that CST prognosis may be ascribable, in part, to cognitive ability at enrolment. The clinical implications of identifying prognostic indicators for CST are significant, and could stimulate a rethinking of when CST is used and who it is used for.

### Limitations

The lack of patient and public involvement in the design of this protocol is a potential limitation of this review; however, patients and the public will be involved in the interpretation of the results. A further limitation is the exclusion of studies published in languages other than English, as this could limit generalisability of the results.

## Data Availability

Data sharing is not applicable to this article as no data-sets were generated or analysed during the current study.

## Supplementary material

For supplementary material accompanying this paper visit http://doi.org/10.1192/bjo.2023.46. click here to view supplementary material
