# Supplementary material for: Identifying prognostic indicators for cognitive stimulation therapy for dementia: protocol for a systematic review and individual participant data meta-analysis
Source: BJPsych Open. 2023 Apr 17;9(3):e69. doi: 10.1192/bjo.2023.46 (PMC10134233; doi:10.1192/bjo.2023.46)
Supplement: Supplementary file 1 [file S2056472423000467sup001.docx]

## **SUPPLEMENTARY FILE 1: SEARCH STRATEGRY**

| Library | Date | Records Retrieved |
| --- | --- | --- |
| Embase | 24/02/2023 |  |
| Ovid | 24/02/2023 |  |
| APA Psych Info | 24/02/2023 |  |

**Ovid**

| 1 | Dementia.mp. or exp Dementia, Vascular/ or exp Dementia/ or exp Frontotemporal Dementia/ or exp Dementia, Multi-Infarct/ |
| --- | --- |
| 2. | exp Alzheimer Disease/ or Alzheimer's.mp. |
| 3 | exp Cognitive Dysfunction/ |
| 4 | 1 or 2 or 3 |
| 5 | "Cognitive Stimulation Therapy".mp. |
| 6 | CST.mp. |
| 7 | Psychotherapy.mp. or Psychotherapy, Group/ |
| 8 | psychotherap$.mp. |
| 9 | 5 or 6 or 7 or 8 |
| 10 | 4 and 9 |

**Embase**

| 1 | Dementia.mp. or exp Dementia, Vascular/ or exp Dementia/ or exp Frontotemporal Dementia/ or exp Dementia, Multi-Infarct/ |
| --- | --- |
| 2. | exp Alzheimer Disease/ or Alzheimer's.mp. |
| 3 | exp Cognitive Dysfunction/ |
| 4 | 1 or 2 or 3 |
| 5 | "Cognitive Stimulation Therapy".mp. |
| 6 | CST.mp. |
| 7 | Psychotherapy.mp. or Psychotherapy, Group/ |
| 8 | psychotherap$.mp. |
| 9 | 5 or 6 or 7 or 8 |
| 10 | 4 and 9 |

**PsychINFO**

| 1 | Dementia.mp. or exp Dementia, Vascular/ or exp Dementia/ or exp Frontotemporal Dementia/ or exp Dementia, Multi-Infarct/ |
| --- | --- |
| 2. | exp Alzheimer Disease/ or Alzheimer's.mp. |
| 3 | exp Cognitive Dysfunction/ |
| 4 | 1 or 2 or 3 |
| 5 | "Cognitive Stimulation Therapy".mp. |
| 6 | CST.mp. |
| 7 | Psychotherapy.mp. or Psychotherapy, Group/ |
| 8 | psychotherap$.mp. |
| 9 | 5 or 6 or 7 or 8 |
| 10 | 4 and 9 |
